# A Neural Mechanism of Strategic Social Choice under Sanction-Induced Norm Compliance^1^,^2^,^3^

**DOI:** 10.1523/ENEURO.0066-14.2015

**Published:** 2015-06-24

**Authors:** Aidan Makwana, Georg Grön, Ernst Fehr, Todd A. Hare

**Affiliations:** 1 Laboratory for Social and Neural Systems Research, Department of Economics, University of Zurich, 8006 Zürich, Switzerland; 2 University of Ulm, University Hospital for Psychiatry, 89075 Ulm, Germany

**Keywords:** decision-making, fMRI, functional connectivity, norm compliance, strategy

## Abstract

In recent years, much has been learned about the representation of subjective value in simple, nonstrategic choices. However, a large fraction of our daily decisions are embedded in social interactions in which value guided decisions require balancing benefits for self against consequences imposed by others in response to our choices. Yet, despite their ubiquity, much less is known about how value computation takes place in strategic social contexts that include the possibility of retribution for norm violations. Here, we used functional magnetic resonance imaging (fMRI) to show that when human subjects face such a context connectivity increases between the temporoparietal junction (TPJ), implicated in the representation of other peoples’ thoughts and intentions, and regions of ventromedial prefrontal cortex (vmPFC) that are associated with value computation. In contrast, we find no increase in connectivity between these regions in social nonstrategic cases where decision-makers are immune from retributive monetary punishments from a human partner. Moreover, there was also no increase in TPJ-vmPFC connectivity when the potential punishment was performed by a computer programmed to punish fairness norm violations in the same manner as a human would. Thus, TPJ-vmPFC connectivity is not simply a function of the social or norm enforcing nature of the decision, but rather occurs specifically in situations where subjects make decisions in a social context and strategically consider putative consequences imposed by others.

## Significance Statement

A large fraction of our decisions are embedded in social contexts that require balancing benefits for self against the positive or negative reactions of others in response to our choices. Yet, how the brain computes the value for different courses of action in such choices is unknown. We examined the neurobiological mechanisms underlying strategic social choices in the context of potential retributive punishment. Our findings indicate that there are specific increases in the functional interactions between brain regions previously associated with mentalizing about others’ beliefs and key nodes of the brain’s value computation system during choices in which it is necessary to balance direct personal gains against the likelihood of subsequent norm enforcing punishment by other people.

## Introduction

A large portion of our daily decisions are embedded in social interactions in which the values of different behaviors depend on the behavior of relevant others. Such interactions range from major decisions about whether to apply for a new job and risk upsetting current colleagues to mundane choices about how much to tip the bartender at your preferred pub in order to maintain favored patron status. In these and many other situations, social norm compliance must be considered to avoid peer punishment for norm violations. In all these cases, we need to take the likely reactions of other people into account. However, despite their ubiquity, very little is known about how value computation takes place in contexts where one’s own behavior may trigger subsequent responses that affect subjective values.

As a central component of the brain’s decision circuitry, the ventromedial prefrontal cortex (vmPFC) has been associated with value computation in nonstrategic decision contexts ranging from primary to social rewards for both self and others (Nicolle et al., 2012; Bartra et al., 2013; Clithero and Rangel, 2013) and in choices during competitive games (Hampton et al., 2008; Zhu et al., 2012). In addition, vmPFC lesions have been shown to alter choice behavior under strategic conditions where norm violations can result in retributive punishment (Krajbich et al., 2009). Collectively, these data suggest that vmPFC might compute subjective values in strategic social choices that require balancing personal preferences with predictions about how the reactions of others to norm violations will impact outcomes for self, but this idea has not yet been directly tested. Furthermore, how predictions about the opponents’ behavior enter into vmPFC value computations is unknown. One hypothesis is that such information is provided to vmPFC by regions that are involved in mentalizing about others.

Previous research has shown that inferring another person’s beliefs in order to estimate his probable future actions recruits neural circuits including the temporoparietal junction (TPJ; Saxe and Wexler, 2005; Frith and Frith, 2006; Zhu et al., 2012). Moreover, studies on competitive and cooperative interpersonal games suggest that TPJ encodes information about other players that could be used to guide choices (Behrens et al., 2008; Hampton et al., 2008; Coricelli and Nagel, 2009; Bhatt et al., 2010; Hare et al., 2010; Rilling and Sanfey, 2011; Carter et al., 2012; Morishima et al., 2012; Carter and Huettel, 2013). However, whether information encoded in TPJ is incorporated into vmPFC value signals during social norm enforcement choices is unknown. Therefore, we sought to examine whether TPJ-vmPFC interactions underlie value computations in this type of strategic social choice.

We examined brain activity using functional magnetic resonance imaging (fMRI) during decisions about the division of monetary assets between participants paired with either another human (social treatment) or a computer partner programmed to enforce social norm violations (nonsocial treatment). On each trial, participants had to choose how to divide 100 monetary units between themselves and the partner. However, these monetary allocation decisions were made in two distinct contexts. In the punishment context, the partner could punish perceived violations of the social norm for fairness by paying to reduce the participant’s earnings, whereas in the control condition the partner could not enforce norm compliance through retributive punishment. The combination of these treatments and conditions allowed us to examine brain activity that was specific to choices that were both social and required strategic reasoning to optimize direct monetary gain against the probability of profit-reducing punishments for fairness norm violations.

## Materials and Methods

### Participants

Forty-seven healthy, right-handed male students performed a strategic economic game while undergoing fMRI scanning. Participants were screened for fMRI contraindications including acute medical conditions and psychiatric or neurological illness. All participants provided written informed consent in accordance with the local ethics committee.

### Behavioral Paradigm

The behavioral paradigm proceeded as follows. On each trial, participants split 100 monetary units (MUs) between themselves (Player A) and Player B. For 24 participants Player B represented a human counterpart (social treatment group, mean age ± SD, 23.5 ± 2.3 years) and for 23 participants Player B was a computer (nonsocial treatment group, mean age ± SD, 24.8 ± 1.9 years). Participants were randomly assigned to either the social or nonsocial treatment groups upon arrival for the experiment. One participant from the social treatment was excluded from all analyses for a lack of comprehension of the task and two participants from the nonsocial group were excluded from the fMRI analyses described below because they never transferred any MUs (leaving 23 social and 21 nonsocial participants). The social group was instructed that each human Player B’s punishment decisions had been acquired in a previous experiment using the strategy method. This method involved Player B making a decision about how many monetary units to spend on punishment if Player A transferred a specific amount. The punishment rate selected by human Player Bs decreased with greater transfers in an approximately linear fashion. The data from all Player Bs was used to generate a punishment distribution function and program the computer algorithm for the nonsocial treatment. The nonsocial group participants were instructed that they were playing against a computer that had been programmed to simulate the responses of the previous human Player B group and were given the same details as the social treatment participants about the strategy method of choice elicitation for Player Bs. All participants were randomly matched against different players on each trial (i.e. a one-shot game). Payment included 20 Euros for participating and 1 Euro per 100 MU earned. Each trial consisted of a treatment screen indicating the trial type for 6 s, a participant driven decision period (mean 4.3 s, SD 2.7 s), then a wait period of 6 s followed by a feedback screen displayed for 6 s. Trials were separated by a fixation cross ITI for 6–8.7 s, sampled from a uniform distribution, thus the decision period started at least 12 s after the previous trials feedback. During the task, participants faced 12 control trials (CON) and 12 punishment trials (PUN) in a random order as indicated during the treatment screen. In CON trials, Player B was not able to punish Player A for making a selfish split (i.e. a dictator game scenario); however, in PUN trials Player B could punish Player A by 5 MUs for each 1 MU spent. Both participants began every trial with a reserve of 25 MUs and therefore, Player B was always able to punish Player A completely (i.e. take away all earnings) during the punishment trials.

### Behavioral Analysis

The behavioral variable of interest was the amount kept/transferred by participants in the role of Player A as a function of group and condition. There was a non-normal distribution of transferred amounts in CON trials (Kolmogorov–Smirnoff test, *p* = 0.03), therefore, we analyzed the transfer amount data using nonparametric Wilcoxon signed rank (paired) and Kruskal–Wallis rank sum tests. All *p* values reported are based on two-sided tests. To better describe the punishment distributions, we linearly regressed punishment on the transfer amount for the social and nonsocial groups.

### MRI Acquisition

Blood oxygen level-dependent (BOLD) echo planar imaging (EPI) scans were performed on a 3 Tesla Siemens Magnetom Allegra using 32 slices and a voxel resolution of 2 × 2 × 2 mm (+0.5 mm slice gap), with a TR of 2490 ms, and a TE of 38 ms. All fMRI data was acquired during a single scanning session (mean length of 750 s, SD 38.5 s). A full brain EPI (56 slices using the same parameters as functional EPI) and anatomical scan (sagittal MPRAGE T1 sequence with a voxel size of 1 × 1 × 1 mm) were also acquired. The fMRI data preprocessing included slice-time correction, spatial realignment to the mean EPI image for each subject, normalization to MNI space, and smoothing with a 10 mm FWHM Gaussian kernel using the SPM 8 software (Wellcome Department of Imaging Neuroscience, Institute of Neurology, London, UK).

### fMRI Analysis

Our primary GLM (GLM-1) was computed to examine BOLD activity relating to the amount kept/transferred during the decision period. GLM-1 modeled four regressor types: (1) treatment, (2) decision, (3) wait, and (4) feedback periods in all trials (PUN and CON) and separately for CON only (8 regressor onsets in total). Single 0 s duration stick functions were convolved with the canonical HRF for the treatment, decision and feedback periods, and a 6 s boxcar function was used for convolution during the wait period. In addition, we used three parametric regressors (PR): (PR1) kept amount at decision onset in all trials, (PR2) kept amount at previous within-condition decision, and (PR3) profit amount at feedback onset for all trials. Six motion parameter regressors were also included in GLM-1. Note that the initial endowment is fixed at 100 MUs for every trial, and therefore, a positive correlation with the amount kept by Player A (PR1) implies a negative correlation with amount transferred to Player B.

SPM 8 software was used to estimate GLM-1 and compute contrasts of interest in each individual participant.

At the second level, we used the “randomise” function from the FSL 5.0.6 software package (http://www.fmrib.ox.ac.uk/fsl/) to test for regions that reflected the amount kept across all participants. We computed a one sample t-test on the single participant contrasts for positive correlations with the kept amount regressor together with a nuisance variable (0 = social, 1= nonsocial) to explain variance due to social and nonsocial participant groups. We performed the *t* test using the nonparametric permutation algorithm in randomise in combination with the threshold-free cluster enhancement (TFCE) method implemented in FSL (Smith and Nichols, 2009). Test statistics and *p* values were derived from 5000 permutations. We corrected for multiple comparisons using familywise error correction at the whole-brain level to achieve corrected significance levels of *p* < 0.05.

### PPI Analysis

For each participant, a seed time course in vmPFC was extracted from a 4 mm sphere centered on the voxel with the strongest correlation with kept amount in that participant from within the overlapping voxels for the group vmPFC cluster generated by GLM-1 and an anatomical mask of vmPFC including the rectal gyrus, medial orbitofrontal, and anterior cingulate cortex below *z* = 5 (5464 8 mm^3^ voxels) based on the AAL atlas (Tzourio-Mazoyer et al., 2002). The vmPFC time series was deconvolved as outlined by Gitelman et al., (2003) before creating the psychophysiological interaction regressors. For the psychophysiological interaction (PPI) GLM (GLM-PPI), the vmPFC time series was used as a physiological regressor and interacted with two separate psychological boxcar regressors for the decision period in CON and PUN conditions. This resulted in two separate psychophysiological interaction terms in GLM-PPI. In GLM-PPI, the decision period duration was modeled as 5 s before the first button press. This expanded window was used because the precise timing of the amount to keep/transfer computation within the treatment and decision screen periods cannot be determined in this task. However, this timing resolution limitation would not bias the results in favor of any specific decision type and, if anything, works against the current findings by adding noise to the analysis. GLM-PPI consisted of the following nine regressors: (1) vmPFC time series, (2) CON decision period boxcar, (3) PUN decision period boxcar, (4) CON decision × vmPFC, (5) PUN decision × vmPFC, (6) CON wait period (6 s boxcar), (7) PUN wait period (6 s boxcar), (8) CON profit screen (6 s boxcar), and (9) PUN profit screen (6 s boxcar). Note that, a one-way ANOVA for the SDs of the PPI regressors for group and condition showed that they were not significantly different (*F*_(1,83)_ = 1.14, *p* = 0.338) suggesting that the PPI analysis was not biased against CON conditions where kept amounts showed less variance. Similar to GLM-1, parametric regressors for kept amount at decision, previous kept amount at decision and profit amount at feedback were included for both punishment and control conditions. Last, GLM-PPI included the six motion parameter regressors. A PPI analysis using the dorsomedial prefrontal cortex (dmPFC) seed noted in Table 1 was also performed. The analysis was identical to GLM-PPI, except that the BOLD time courses were extracted from the dmPFC ROI rather than the vmPFC ROI described above.

**Table 1:** Regions correlating with the amount kept by Player A at the time of choice

Region	Hemisphere	Extent	*x*	*y*	*z*	Peak T
Lingual gyrus	R/L	1257	8	−74	8	5.21
Cingulate gyrus	R/L	39	2	−10	36	4.54
vmPFCa-ACC	R/L	36	0	48	−2	4.14
mPFC-paracingulate gyrus	R/L	30	0	54	4	3.79
mPFC-ACC	R/L	28	−4	44	14	3.91
Frontopolar cortex/IFG	R	23	42	44	0	4.75
dmPFCb-paracingulate/SFG	R/L	21	−2	50	26	5.45
Occipital cortex	R	20	42	−76	−6	4.69
ACC	R/L	18	0	26	28	4.22
Thalamus	L	16	−12	−34	8	3.90
vmPFC-ACC	R	14	8	48	0	3.82
Frontopolar cortex	L	11	−16	58	28	5.00
Cingulate gyrus	L	10	−4	−4	32	3.95
						

Peak coordinates (*x*,*y*,*z*) are listed in MNI space. T values are test statistics derived from 5000 permutations of the data. All regions are significant at *p* < 0.05 whole-brain familywise error corrected for multiple comparisons.

IFG, Inferior frontal gyrus; SFG, superior frontal gyrus; R, right; L, left.

*^a^*vmPFC cluster used as a mask to extract subject specific time courses for PPI analyses.

*^b^*dmPFC cluster used as a mask to extract subject specific time courses for PPI analyses.

Following estimation of GLM-PPI in SPM8, single participant contrasts were computed for regressors of interest. At the second level, we again used TFCE and the nonparametric permutation function, randomise, to test for between group differences in connectivity with vmPFC. Test statistics and *p* values were derived from 5000 permutations. Based on previous work (Morishima et al., 2012) showing that social preferences during interpersonal interactions are linked to structural and functional differences in the TPJ, we created a spherical ROI with 10 mm radius around the MNI coordinates (*x*, *y*, *z* = 60, −44, 18). The conjunction of this ROI and the group functional coverage mask was used for small volume correction (324 8 mm^3^ voxels). This functional coverage map was utilized because the acquisition parameters for the functional MRI data did not provide whole-brain coverage, and in some cases, the tilt of the transverse slices relative to anterior commissure–posterior commissure resulted in lack of coverage for the superior temporal and inferior parietal cortex. Forty-two participants (21 social and 21 nonsocial) had adequate functional coverage and were included in the PPI analysis. We corrected for multiple comparisons using familywise error correction within this mask to achieve small volume correction (SVC) of *p* < 0.05.

The bar plots shown in Figure 3*c* were created by taking the average vmPFC- TPJ PPI coefficients from all voxels in the functional ROI for the difference between social and nonsocial punishment trials shown in Figure 3*a*. These bar plots are presented for visualization purposes only and were not used as a basis for any statistical analysis.

In addition to comparing the PPIs during the PUN decisions between groups, we also tested for an association between the vmPFC-TPJ PPI during punishment decisions and the average punishment received by each individual within the social and nonsocial groups. We applied the same TPJ small volume correction described above for this analysis.

Last, we performed a *post hoc* analysis of correlations with profit during the PUN feedback condition (GLM-PPI regressor 9) by extracting PUN profit betas from all significant voxels in the social PUN PPI cluster shown in Figure 3*b*.

## Results

**Figure 1:**
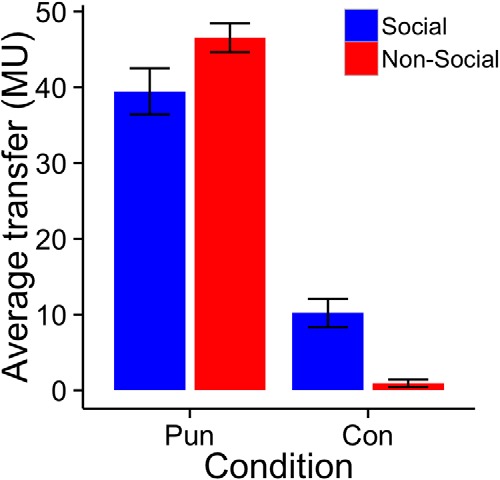
Amounts transferred by Player A in the PUN and the CON condition of both the social and the nonsocial treatment. Transfers are represented in experimental monetary units out of a given amount of 100 units. Error bars represent the standard error of the mean for the group mean. Paired sample Wilcoxon signed rank tests (social W = 276, *p* = 2.88e−5; nonsocial W = 231, *p* = 6.36e−5) showed significant differences between the PUN and CON transfer rates in each group.

**Figure 2:**
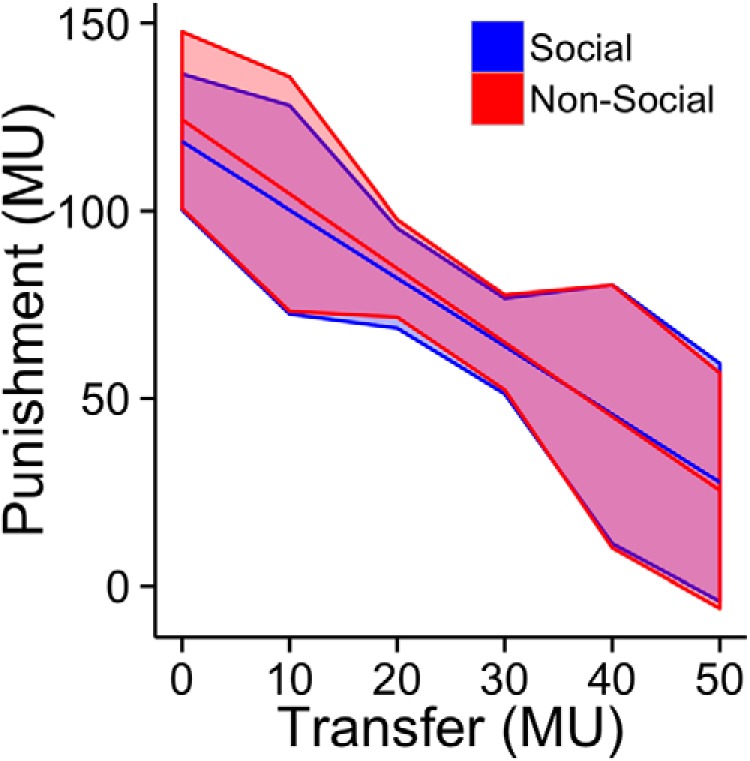
The plot shows punishment distributions as a function of amount transferred for both social (blue) and nonsocial groups (red). Punishment was regressed onto transfers up to 50 MUs, with the predicted punishment (thick line) and the SDs of the residuals (shaded area) for each transfer amount. Transfers >50 MU resulted in zero punishment. The overlapping distributions for the social and nonsocial treatments indicate that the computer algorithm was successful in replicating human punishment behavior.

Behaviorally, there was no difference in the total amounts transferred between the social and nonsocial treatment groups (Kruskal–Wallis *Χ* ^2^_(1,_*_N_*_=88)_ = 0.48, *p* = 0.49). Transfers in the social CON condition were on average 9.3 MU (SD 17.0), leading to an average percentage split of 22.9% for Player B after accounting for the 25 MU reserve amount for both players. These transfer rates are consistent with average rates (∼20%) reported in the previous literature (Camerer, 2003). Participants in the role of Player A transferred more in PUN than CON conditions in both the social [Wilcoxon signed rank (W) = 276, *p* = 2.88e−5] and the nonsocial treatments (W = 231, *p* = 6.36e−5; Fig. 1). These results suggest that Player A strategically increased the amount transferred to Player B to decrease the likelihood that Player B would exercise his punishment option and reduce Player A’s earnings regardless of whether Player B was a human or a computer programmed to mimic human reactions. Increasing the amount transferred in PUN trials was in fact the best strategy for Player A to maximize his earnings because the punishment amount decreased with greater transfers (with zero punishment above a transfer of 50 MUs) in an approximately linear fashion (Fig. 2).

**Figure 3:**
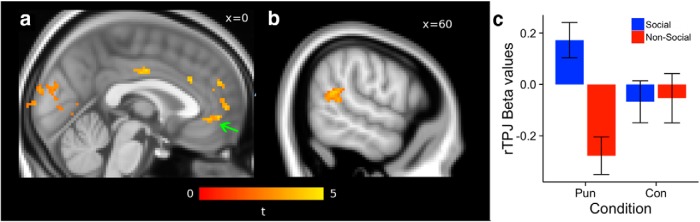
Activity and connectivity at the time of choice. ***a***, Regions showing a positive correlation with the amount of monetary units participants decided to keep on each trial across all decision types. The green arrow indicates the vmPFC cluster used to extract time courses for the PPI analysis. ***b***, Region of TPJ showing increased functional connectivity with vmPFC during strategic decisions made in social punishment compared with nonsocial punishment contexts. All voxels shown in ***a*** and ***b*** are significant at *p* < 0.05 after correcting for multiple comparisons. ***c***, Bar graph showing the relative coupling between vmPFC and TPJ by treatment group and choice context and demonstrating that increased TPJ-vmPFC coupling is specific to choices that are both strategic and social in nature. Error bars represent the standard error of the mean for the group mean. These bar plots are presented for visualization purposes only and were not used as a basis for any statistical analysis.

In our initial neuroimaging analysis, we examined the degree to which vmPFC activity reflected value computations during monetary transfer decisions in both treatment types using a general linear model on BOLD signals. This analysis showed a positive association between kept amounts and vmPFC BOLD activity (Fig. 3*a*; *p* < 0.05 whole-brain corrected) across all participants. In addition to vmPFC, BOLD activity in dmPFC, right frontopolar cortex, and occipital regions also correlated with the amount kept at the time of choice (Table 1). The correlation between amount kept and BOLD activity in the vmPFC ROI was not significantly different between treatment groups (two-sample *t* test, *t*_(42)_ = 1.4, *p* = 0.332 uncorrected) indicating that participants playing against humans and computers represented the amount kept to an equal degree in vmPFC. Furthermore, there was no significant difference between the social and nonsocial groups in the correlation with amount kept and BOLD activity in any brain region after correcting for multiple comparisons.

The vmPFC result is consistent with theoretical models and existing empirical data suggesting a central role for vmPFC in the computation of subjective values for a wide range of decision contexts (Kable and Glimcher, 2009; Rangel and Hare, 2010; Rushworth et al., 2012; Bartra et al., 2013; Clithero and Rangel, 2013). Such theories also posit that if vmPFC acts as a general valuation system, then its interactions will be modulated such that coupling with regions providing decision relevant information will increase.

Next, we tested the hypothesis that the coupling between vmPFC and the right TPJ will increase more during decisions that require strategic evaluations of another person’s response to the outcome than in complexity matched control conditions using a PPI analysis with the vmPFC as the seed region. This analysis examines whether the correlations between vmPFC activity and other brain regions differ in social versus nonsocial PUN transfer decisions. Note that in both the social and nonsocial PUN conditions participants need to make strategic transfer decisions that take into account Player B’s likely level of punishment (i.e. fairness norm enforcement), and it is only the nature of Player B (human vs computer) that differs between groups. We found that participants in the social treatment showed more positive correlations between TPJ and vmPFC in PUN trials compared with the nonsocial treatment (Fig 3; *p* < 0.05 SVC; peak T = 3.97 at x, y, z = 60, −48, 16; extent = 115 voxels). *Post hoc* one-sample *t* tests showed that the average PPI effect in these voxels for social PUN was greater than zero (*t*_(20)_ = 2.51; *p* = 0.021), whereas the average PPI effect for nonsocial PUN was less than zero (*t*_(20)_ = −3.79; *p* = 0.001). Exploratory analyses revealed no other regions that showed this pattern of connectivity with vmPFC after correcting for multiple comparisons. However, for completeness, we also list regions exceeding a threshold determined by the lowest individual voxel *t* statistic (*t* > 2.29) derived from the right TPJ cluster (Table 2). Furthermore, there were no voxels that showed a significant PPI effect in either social or nonsocial CON trials after correcting for multiple comparisons within the independent TPJ ROI or in the entire volume.

**Table 2:** Location and extent of functional clusters showing a difference in PPI with vmPFC between social and nonsocial PUN decisions that was greater than or equal to the effect in our *a priori* TPJ region

Region	Hemisphere	Extent	*x*	*y*	*z*	Peak T
TPJ	R	144	60	−48	16	3.97
Parahippocampal gyrus	R	93	22	−26	−14	3.87
Lingual gyrus	R	86	28	−50	4	3.87
Fusiform cortex	L	78	−34	−38	−18	4.12
Fusiform cortex	L	66	−38	−2	−32	4.31
White matter/insular cortex	L	60	−28	−16	24	3.96
STG	L	51	−52	−24	6	3.36

Peak coordinates (*x*,*y*,*z*) are listed in MNI space. T values are test statistics derived from 5000 permutations of the data. Clusters reported are all of those that surpass a threshold set by lowest *t* value in the small volume corrected TPJ cluster (*t* > 2.29) and minimum cluster size of 50 voxels (2 × 2 × 2 mm). Note that these results are reported here for completeness only and are not corrected for multiple comparisons and thus not the subject of any inference in this paper. STG, Superior temporal gyrus.

**Figure 4:**
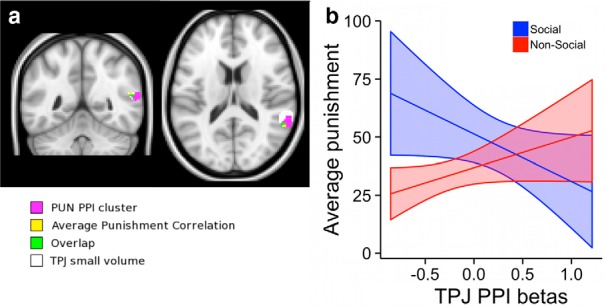
Regions of the TPJ relating to the vmPFC PPI at time of choice. ***a*,** The voxels in magenta show greater increases in connectivity with vmPFC during PUN choices in the social compared with the nonsocial group, controlling for connectivity in CON trials. Voxels in yellow are those where the PUN − CON PPI was significantly correlated with individual average punishment levels in the social, but not the nonsocial group. Green voxels represent the overlap of both effects. Clusters are significant at *p* < 0.05 SVC within the TPJ small volume shown in white. ***b***, The fitted regression slopes between TPJ-vmPFC PPI at the time of choice and an individual’s average punishment level separately for the social (blue) and nonsocial (red) groups. The shading around the regression lines indicates the 95% confidence intervals.

To test whether vmPFC-TPJ PUN PPI strength is related to the overall strategic play of the participants, we tested whether the individual PPI difference contrast (PUN − CON) differentially correlated with participants’ average punishment amounts in the social compared with nonsocial groups. This second level, between subjects regression analysis revealed a link between vmPFC-TPJ PPI during PUN trials and average punishment levels that were stronger in social more than nonsocial treatment participants. In the social group, greater vmPFC-TPJ PPI was associated with less punishment by Player B, whereas there was no significant relationship in the nonsocial group (Fig. 4; *p* < 0.05 SVC; peak T = 3.88 at *x*, *y*, *z* = 56, −50, 16; extent 8 voxels).

For completeness, we repeated our PPI analysis replacing the vmPFC seed with a region of dmPFC that also correlated with amount kept at the time of choice. We tested this dmPFC seed in addition to vmPFC because the dmPFC has been implicated in alternative value representation and strategic mentalizing processes (Frith and Frith, 2006; Hampton et al., 2008; Coricelli and Nagel, 2009; Nicolle et al., 2012; Zhu et al., 2012). However, we found no significant differences in connectivity with the dmPFC during social compared with nonsocial PUN trials within our TPJ ROI or at the whole-brain level after correcting for multiple comparisons.

We also examined brain activity at the time of outcome when subjects learned how much profit they had made in the previous trial. We found that the parametric regressor for profit magnitude at outcome (PR3 from GLM-1) correlated with BOLD activity in several regions, including bilateral striatum and left lateral frontal cortex (*p* < 0.05 whole-brain corrected; Table 3). Just as with the BOLD correlations at the time of choice, there were no regions showing a difference between the social and nonsocial groups in the correlation with profit magnitude at outcome. In addition, we conducted an ROI analysis on the BOLD correlation with profit at feedback using the voxels from the TPJ cluster showing the PUN PPI difference between the groups. We found that across all subjects there was a significantly negative effect of profit on TPJ activity at the time of feedback (one sample *t*_(41)_ = −2.15, *p* = 0.037), and once again, the groups did not significantly differ in this effect (two sample *t*_(40)_ = 0.13, *p* = 0.90).

**Table 3:** Regions correlating with profit at the time of feedback

Region	Hemisphere	Extent	*x*	*y*	*z*	Peak T
Insula/striatum	R/L	1645	32	12	4	6.97
Striatum	L	475	−30	−14	10	5.5
Frontopolar cortex	L	325	−38	60	4	4.88
Precentral gyrus	R	44	58	−4	22	4.17
Caudate tail	R	16	18	−4	26	3.85
Posterior insula	L	16	−38	−18	0	4.32
Parietal operculum	L	10	−48	−30	22	4.28

Peak coordinates (*x*,*y*,*z*) are listed in MNI space. T values are test statistics derived from 5000 permutations of the data. All regions are significant at *p* <0.05 whole-brain familywise error corrected for multiple comparisons.

## Discussion

Our results indicate a role for vmPFC in the computation of value during strategic choices involving norm enforcement and suggest that increased TPJ-vmPFC coupling is especially important in decisions that involve strategic considerations of how social others will react to one’s own actions. Despite the fact that participants were fully informed that the computer opponents were programmed to punish fairness norm violations at the same levels as real human players, the coupling between TPJ and vmPFC value computation regions did not increase in nonsocial PUN decisions, and in fact, decreased relative to the nondecision baseline.

This pattern of TPJ results is consistent with previous experiments showing that multivariate analyses of TPJ activity could be used to help predict bet and fold decisions in a simplified poker game against human opponents, but including TPJ activity measures actually decreased the model’s predictive power for computer opponents (Carter et al., 2012). These previous experiments did not however examine the connectivity between TPJ and other brain regions. Our TPJ-vmPFC connectivity results demonstrate that in the realm of value-based choices, TPJ-vmPFC coupling increases during strategic choices when paired with human counterparts, but decreases with computer partners. Moreover, increased connectivity between vmPFC and TPJ at the time of choice is associated with more advantageous strategic decision-making (i.e. lower norm-enforcing punishment) in social but not nonsocial contexts. This is consistent with the idea that vmPFC incorporates information from distributed brain regions into value computations and that inputs are either enhanced or inhibited as a function of their relevance in the current state. Moreover, TPJ-vmPFC coupling did not significantly increase in either the social or the nonsocial CON trials where punishment predictions were not necessary because the opponent could not respond. This indicates that TPJ-vmPFC connectivity was not simply a function of the social nature of the decision, but rather occurred selectively when both social and strategic factors were in play.

Previous work has shown that TPJ activity reflects social learning signals in the context of repeated interactions where it is advantageous to learn about other human players (Behrens et al., 2008; Hampton et al., 2008). This learning takes the form of update signals measuring deviations from the expected result at the time of feedback when decision outcomes are shown. These update or error signals are presumably used to guide subsequent choices when paired with the same person in the future, although the impact of TPJ activity at the time of subsequent choices was not explicitly examined in these previous reports. In the current paradigm, participants are paired with a different human partner on each trial, and therefore, outcomes of previous trial choices cannot be directly applied to future decisions. However, it may be that TPJ activity also plays a role in forming expectations based on average or normative behavior. There is a strong social norm for fairness and this norm could be used as a basis for predicting the degree of punishment by an unknown Player B that would result from various monetary splits. Consistent with this role, we found that TPJ activity increased when participants were shown feedback indicating that a strategic adjustment was necessary (i.e. low profits) on the following choice to avoid future norm enforcing responses from Player B. Moreover, the results summarized in Figure 4 suggest that increased connectivity between TPJ and vmPFC may be a mechanism by which such predictions are incorporated into value computations at the time of choice.

In addition to vmPFC, BOLD activity in several other brain regions, particularly dmPFC, correlated with the amount kept for oneself when deciding how to allocate MUs on each trial. The correlation with kept amount in dmPFC is of particular interest given previous findings that activity in this region relates to individual differences in type or level of reasoning during social interactions (Hampton et al., 2008; Coricelli and Nagel, 2009; Zhu et al., 2012). Our findings in dmPFC build on these previous individual difference results and demonstrate that this region also reflects choice specific components of strategic valuation during decisions in which social norm compliance can be enforced through peer punishment. Although the current dataset was not designed to distinguish between the value related activity in regions such as vmPFC and dmPFC, previous reports have suggested that there is a dorsal to ventral gradient for modeled and executed value functions along the mPFC (Nicolle et al., 2012). If our subjects are engaging in predictive forecasting (i.e. modeling) of Player B’s responses to their transfers and decisions are taken (i.e. executed) on the basis of these models, then this could explain why we find activity correlated with the amount kept in both ventral and dorsal portions of mPFC. However, further experiments will be necessary to test this speculative hypothesis.

One limitation of the current dataset is that there were a relatively small number of choices for each participant per condition (*n* = 12). Therefore, it is possible that future studies including more choices per participant, and thus having greater power, will find additional changes in vmPFC connectivity associated with social strategic decision-making.

Decisions that balance welfare for self with the impacts on and reactions of others to one’s own choices are ubiquitous in social life. Our results provide insights into the neural mechanisms underlying such behavior and suggest a key role for interactions between TPJ and vmPFC. These findings are an important advance in our understanding of the neurobiology underlying strategic social choice and provide a basis for future investigations into this central aspect of human behavior.

## Figures and Tables

**Figure:**
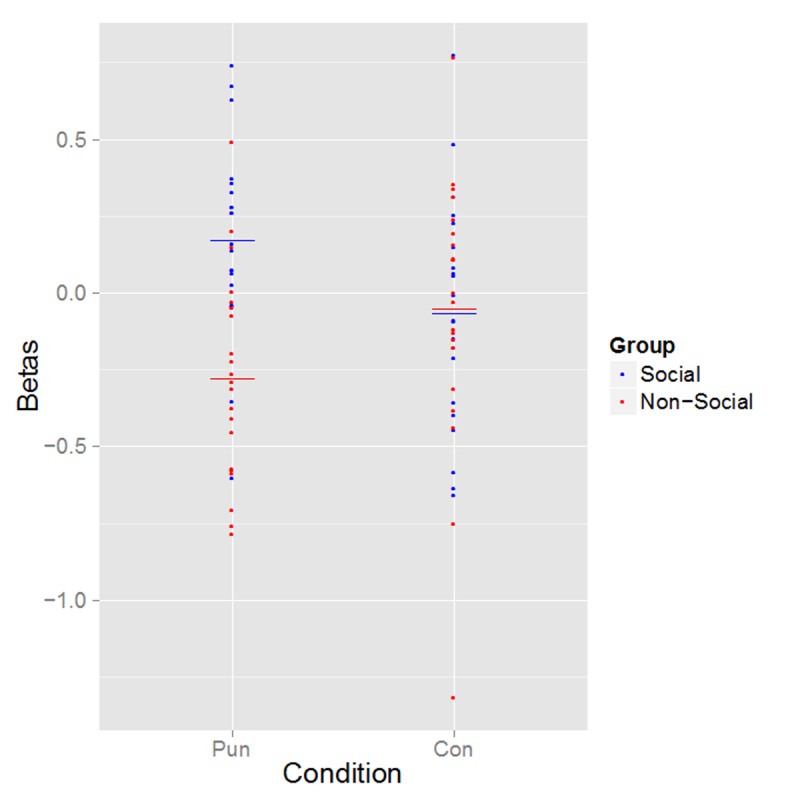


## Synthesis

The decision was a result of the Reviewing Editor Lila Davachi and the peer reviewers coming together and discussing their recommendations until a consensus was reached. A fact-based synthesis statement explaining their decision and outlining what is needed to prepare a revision is listed below.

### Synthesis of Reviews:

Both reviewers feel that the question of the current manuscript - asking how value computations may differ in social contexts - is timely and important and that the results are potentially interesting. However, both reviewers suggest that that the result is ambiguous without further contemplation and analysis of your data. Specifically, if the specificity of the main finding (connectivity between TPJ and vmPFC) can be further queried to isolate specific computations that may be supported by TPJ then the manuscript can be further considered. Furthermore, there were a number of methodological details that need to be clarified/included in a revised manuscript. Finally, the main connectivity result rests on connectivity computed across 12 trials, which is a very small sample. My main concern with this is that one or two outlier trials could greatly skew the correlation in any one subject. Have measures been taken to eliminate outlier beta parameter estimates? If you feel you can address these concerns and provide the additional data requested in addition to addressing each of the other concerns of the reviewers, then we would be willing to consider a revision.

### Specific Comments of Reviewers:

The current study investigated the neural substrates of value computation in strategic social contexts, defined as decisions where consequences are determined by one's own actions as well as action of others. Using an economic game paradigm, the authors found that in social interactions that involve strategic motivations, there was an increase in effective connectivity between TPJ and vmPFC, as compared to conditions where either social and strategic components were missing.

The results of this study has potentially important implications for our knowledge of the neural basis of social decision-making and valuation in general. Despite some strengths, there are a number of important issues at the methodological and expositional levels that prevent me from recommending this paper.

1. There are a number of missing details that raise important questions about both behavior and neural data, as well as their suitability for the present research question. For example, is there enough variation in choice behavior in the control conditions to believe that the dissociation between PUN and CON constitute a strong test of dissociation? This is particularly salient for the CON conditions, where the transfer rates are exceedingly low.

The low transfer rates in the social CON trials is also quite puzzling from a behavioral standpoint, given that it is essentially a dictator game. Yet the average transfer is 10, substantially lower than typical dictator game behavior. This is not a problem per se, but does raise questions about what cognitive processes are affected in these experimental manipulations in addition to the "social" dimension that the authors propose.

2. Similar to the behavioral results and analyses, important details of the neuroimaging analyses are either missing or glossed over. For example, this appears to be a very small number of trials for a functional connectivity study (12 trials in each condition for each subject), which deserves some discussion. This is less of a problem for studies focusing on main effects, but I am less confident in the case of connectivity studies.

3. A key claim of the paper is that the functional connectivity result sheds light on mentalization processes in TPJ affects value computation processes in vmPFC. However, there was little effort made to rule out alternative hypotheses, nor in additional analyses to bolster the current indirect inference. For example, is it possible that differences between social and non-social conditions arise from instructional or expectation differences, which may or may not have a social aspect per se. In addition, can the authors provide more direct evidence that the current TPJ is involved in mentalization or other social inference processes in the game. Previous studies, including Hampton et al. used a modeling approach to isolate computational signatures in TPJ/STS activity. Given the use of a similar interactive game, perhaps the authors can use a similar approach to clarify the nature of the functional connectivity.

Rev 2:

The authors of the present article target the role of value computation in strategic decision-making, focusing on the relationship between vmPFC and TPJ. Using an economic game, they vary whether the participant is playing with another person or a computer, and whether their partner can respond to their offer with punishment or not. They first identify a region in vmPFC that tracks with how many MUs the participant kept on each trial, and then show that connectivity between this region and TPJ is increased in social contexts under the threat of punishment and inhibited when no punishment is possible (with no difference in non-social contexts). The authors conclude that vmPFC selectively incorporates information about others' thoughts when relevant to the decision at hand and inhibits this information when it is not needed.

On the whole, this works contributes to an understanding of how interactions between prefrontal and parietal regions impacts decision making in social contexts. However, I feel that there are a few areas requiring improvement before this work can be considered complete.

Principally, the authors' conclusion that the role of TPJ is in "forming expectations based on average or normative behavior" (lines 323-324) is open to interpretation. There are two possibilities that the authors should address. First, Player B responses (in the social condition) were collected offline and before the task. Is it possible that this impacted the way in which vmPFC and TPJ interacted? Potentially, as participants were deciding whether to transfer money or not, they used ToM to project into the past to cognize about decisions made previously. This result would be in line with the prospection theory of the default model network and theory of mind regions, including the TPJ. If so, this would suggest that coupling between vmPFC and TPJ in this case is not about incorporating others' minds and preferences into strategic decision-making per se, but rather about think about decisions made in the past (as opposed to computations being performed in the present by a computer). Do the authors have evidence against this possibility? Second, more description of the behavior of Player B is needed. How did these participants punish? Was there in fact a norm present that participants had to learn? Or was behavioral wildly divergent? Answers to these questions bear on whether participants did indeed need to think about others' preferences and thus the computational role that TPJ is playing.

There were also a few minor issues:

- The authors' uncorrected thresholds (p0.001 k=10) were actually quite restrictive, given the small number of trials per subject and the general lack of activation seen in the main GLM. Exploratory analyses at a more lenient threshold, potentially p 0.01 k = 10 or similar, would shed light on whether vmPFC activity during strategic decision-making is influenced by regions other than TPJ.

- More details are needed about data acquisition. How many runs were acquired and how many trials per run? Similarly, how were trials in the task distributed? Did they vary randomly or were they blocked?

- On line 111, the authors indicate that "Trials were separated by a fixation cross ITI for approximately 6s." More information would be useful here. Given such a short ITI, the HRF would not have returned to baseline levels by the start of the next trial type screen (which was included in the decision period regressors in the PPI). Thus, unless jittering was included, activity from the previous trial's feedback phase could be impacting the PPI analysis. This may impact how coupling between vmPFC and TPJ is interpreted.

- Line 216: "Figure 3b" should instead be "Figure 2c"

- Line 263: "Fig 3" should be "Fig. 2"

Editor Synthesis of Reviews:

Both reviewers feel that the question of the current manuscript - asking how value computations may differ in social contexts - is timely and important and that the results are potentially interesting. However, both reviewers suggest that that the result is ambiguous without further contemplation and analysis of your data. Specifically, if the specificity of the main finding (connectivity between TPJ and vmPFC) can be further queried to isolate specific computations that may be supported by TPJ then the manuscript can be further considered. Furthermore, there were a number of methodological details that need to be clarified/included in a revised manuscript. Finally, the main connectivity result rests on connectivity computed across 12 trials, which is a very small sample. My main concern with this is that one or two outlier trials could greatly skew the correlation in any one subject. Have measures been taken to eliminate outlier beta parameter estimates? If you feel you can address these concerns and provide the additional data requested in addition to addressing each of the other concerns of the reviewers, then we would be willing to consider a revision.

-We were happy to read the reviewers' positive opinions on the importance and timeliness of our work and also grateful for the suggestions for improvement made by both reviewers and the editor. We have conducted several new analyses based on these suggestions and believe that they serve to considerably strengthen the impact of the paper. We will briefly respond to the editor's summary here and then address each reviewer's specific points in detail below. The original comments are in black font and our replies in blue to easily distinguish the two. First, with regard to the potential computations supported by the TPJ in this paradigm, we have two new findings that provide additional insight into its role in strategic social choice. Specifically, we now show that:

1) Right TPJ activity is sensitive to outcome feedback indicating a need for adjustments in behavior to avoid future norm enforcing punishments.

2) Individual differences in the level of TPJ-vmPFC PPI at the time of choice are correlated with the total profit made (i.e. strategic choice success) and that this relationship is specific to the Social treatment.

This second new result above also has direct implications for concerns expressed by the editor and Reviewer 1 about the number of decisions included in our PPI analysis. The significant relationship between TPJ-vmPFC PUN PPI and total profit indicates that we can estimate the PPI beta parameters with sufficient precision to capture individual differences in behavior. However, we also sought to satisfy the request to check for the presence of extreme PPI beta parameter estimates in any of our participants. In the figure below, we plot the individual PPI beta parameters for all participants in the Social (blue) and Non-social (red) treatments. This plot shows that there is a separation between treatment groups during PUN, but not CON decisions and that the group difference in mean PUN TPJ-vmPFC PPI is not driven solely by extreme values in a single or a small number of participants.

Lastly we have clarified or added all methodological details requested by the reviewers.

REBUTTAL LETTER FIGURE GOES HERE

This figure plots the vmPFC-TPJ PPI betas for each participant in the Social (blue) and Non-social (red) groups. Betas represent the average across all voxels of the ROI depicted in Figure 3b of the main text during both PUN and CON decisions. Horizontal lines represent the group means in each condition.

### Reviewer 1:

The current study investigated the neural substrates of value computation in strategic social contexts, defined as decisions where consequences are determined by one's own actions as well as action of others. Using an economic game paradigm, the authors found that in social interactions that involve strategic motivations, there was an increase in effective connectivity between TPJ and vmPFC, as compared to conditions where either social and strategic components were missing.

The results of this study has potentially important implications for our knowledge of the neural basis of social decision-making and valuation in general. Despite some strengths, there are a number of important issues at the methodological and expositional levels that prevent me from recommending this paper.

1. There are a number of missing details that raise important questions about both behavior and neural data, as well as their suitability for the present research question. For example, is there enough variation in choice behavior in the control conditions to believe that the dissociation between PUN and CON constitute a strong test of dissociation? This is particularly salient for the CON conditions, where the transfer rates are exceedingly low.

-The reviewer raises an important concern about variability in the CON decision trials. There are two relevant sources of variation to consider, the first is behavioral variation in amount transferred and the second is the variation in vmPFC activity during the psychophysiological interaction window. Although there is less variability in the amount transferred during CON trials, there is still substantial variation in both the Social PUN and Social CON decisions that we wish to dissociate, 410 MU2 and 309 MU2, respectively. Similar levels of variation are also present in Non-social PUN transfers 314 MU2. As expected there is little variance in the Non-social CON trials in which participants would be giving away money to a computer (22 MU2). Overall, participants' transfer behavior reflects the social preferences and strategic concerns present in each decision condition. The second aspect of variation that is arguably most important for the PPI analysis at the core of our paper is the level of activity in vmPFC during PUN and Con choices. Because the psychological regressors simply mark the onsets in each condition and thus contain the same variance for all conditions, any difference in the variability in the PPI regressors is driven by the vmPFC BOLD time series. We found that there is no difference in the standard deviations of the PPI regressors across the four conditions (F1,83 = 1.14, p = 0.338), and thus this issue is not a concern when interpreting the PPIs in any condition. This result is reported on page 9, lines 200-202 in the current manuscript.

The low transfer rates in the social CON trials is also quite puzzling from a behavioral standpoint, given that it is essentially a dictator game. Yet the average transfer is 10, substantially lower than typical dictator game behavior. This is not a problem per se, but does raise questions about what cognitive processes are affected in these experimental manipulations in addition to the "social" dimension that the authors propose.

-The reviewer is correct that an average transfer rate of 10 out of 100 would be lower than the ∼ 20% rate that is often seen in the literature (see for example Colin Camerer's 2003 book, "Behavioral Game Theory: Experiments in Strategic Interaction"). However, there is an additional factor to consider in our version of the game, that being the reserve amount of 25 MUs. The reserves add 25 MU to each player's payout in the dictator game, and therefore, a transfer of 10 is approximately 23% of the total. This percentage transfer is similar to the levels reported in previous research, although there is some variation from study to study based on how the game is presented (e.g. List 2007 Journal of Political Economy). We have modified the text to highlight that the presence of the monetary reserve in our games is an important difference from previous dictator games. The added text begins on page 11, line 247 of the current manuscript and is copied below for convenience.

"Transfers in the social CON condition were on average 9.3 MU (SD 17.0), leading to an average percentage split of 22.9% for player B after accounting for the 25 MU reserve amount for both players. These transfer rates are consistent with rates (∼20 %) reported in the previous literature (Camerer, 2003)."

2. Similar to the behavioral results and analyses, important details of the neuroimaging analyses are either missing or glossed over. For example, this appears to be a very small number of trials for a functional connectivity study (12 trials in each condition for each subject), which deserves some discussion. This is less of a problem for studies focusing on main effects, but I am less confident in the case of connectivity studies.

-Both reviewers have pointed out that the manuscript would benefit from the addition of specific details regarding the behavioral task and neuroimaging data acquisition and analysis. We have added in all requested information. With regard to the specific point about power and the PPI analysis, the reviewer is correct that there are only 12 trials per condition for each participant (this fact is stated explicitly on page 6, line 122). Trial number is an important issue because fMRI has an inherently low signal to noise ratio and the power to find effects generally increases as a function of the number of repeated measures. A relatively small number of trials would limit the precision with which we can estimate contrasts (both main effects and interactions) at the individual or first level in common fMRI terminology. However, in this case, the small number of trials is offset in part by the 5s boxcar function used for the psychological main effect in our PPIs, making our analysis more like a block design (more efficient and powerful) than a fast event related design. Moreover, and most importantly, any noise in the estimation of first level contrasts would only work against us when examining consistent effects across participants at the second or random effects level. Random effects inference is our primary focus here, just as it is in the vast majority of fMRI studies, because we want to be able to generalize the results to the larger population. The random effects level statistical analysis on a relatively large sample of 42 participants tells us that there is a difference between vmPFC PPIs with TPJ for Pun decisions in social and non-social conditions. However, it is possible that the including more than 12 decision trials per condition would lead to the discovery of additional significant effects beyond our TPJ results. To address the reviewer's concern we have added a statement to the manuscript acknowledging that an experiment with more decision trials per condition might find additional differences in vmPFC PPI beyond those identified in the TPJ. The new text is on page 17, line 400 and is also copied below.

"One limitation of the current dataset is that there were a relatively small number of choices for each participant per condition (n=12). Therefore, it is possible that future studies including more choices per participant, and thus having greater power, will find additional changes in vmPFC connectivity associated with social strategic decision making."

3. A key claim of the paper is that the functional connectivity result sheds light on mentalization processes in TPJ affects value computation processes in vmPFC. However, there was little effort made to rule out alternative hypotheses, nor in additional analyses to bolster the current indirect inference. For example, is it possible that differences between social and non-social conditions arise from instructional or expectation differences, which may or may not have a social aspect per se.

-The instructions for both groups were identical except for the additional explanation of the computer's behavior given to the non-social group. The instructions for the non-social group explained that the computer would draw punishment levels from a distribution derived from actual responses collected from real people. We have added a new figure (Fig. 2) showing that the punishment distributions in the Social and Non-social treatments do not differ.

In addition, can the authors provide more direct evidence that the current TPJ is involved in mentalization or other social inference processes in the game. Previous studies, including Hampton et al. used a modeling approach to isolate computational signatures in TPJ/STS activity. Given the use of a similar interactive game, perhaps the authors can use a similar approach to clarify the nature of the functional connectivity.

-We have conducted additional analyses to support our claims that TPJ activity reflects components of the task that are directly relevant for strategic play and that TPJ-vmPFC connectivity is specifically relevant during decisions with a human partner. The precise modeling approach used in Hampton et al. cannot be used in this experiment because the participants cannot exert influence over their opponents, but can only learn the population distribution of punishments. However, we have carried out new analyses at the time of feedback in order to examine the BOLD correlation with profit (kept amount minus punishment level) in the TPJ ROI presented in the main text, and found that across the whole sample there was a significantly negative correlation with profit (t = -2.15, p = 0.037). Thus, lower profits indicating a need to adjust one's strategy when determining future transfer/kept amounts were associated with higher levels of TPJ activity. This finding suggests that the information relevant for strategic adjustments correlates with TPJ activity during feedback in this task consistent with previous reports of TPJ activity by Hampton et al., 2008 and Behrens et al., 2008.

In addition, we also computed a new analysis testing whether TPJ-vmPFC PPI at the time of choice was related to individual differences in strategic decision making success (i.e. amount of profit earned). We found that increased TPJ-vmPFC coupling leads to better performance in the social, but not the non-social group.

### Reviewer 2:

The authors of the present article target the role of value computation in strategic decision-making, focusing on the relationship between vmPFC and TPJ. Using an economic game, they vary whether the participant is playing with another person or a computer, and whether their partner can respond to their offer with punishment or not. They first identify a region in vmPFC that tracks with how many MUs the participant kept on each trial, and then show that connectivity between this region and TPJ is increased in social contexts under the threat of punishment and inhibited when no punishment is possible (with no difference in non-social contexts). The authors conclude that vmPFC selectively incorporates information about others' thoughts when relevant to the decision at hand and inhibits this information when it is not needed.

On the whole, this works contributes to an understanding of how interactions between prefrontal and parietal regions impacts decision making in social contexts. However, I feel that there are a few areas requiring improvement before this work can be considered complete.

Principally, the authors' conclusion that the role of TPJ is in "forming expectations based on average or normative behavior" (lines 323-324) is open to interpretation. There are two possibilities that the authors should address. First, Player B responses (in the social condition) were collected offline and before the task. Is it possible that this impacted the way in which vmPFC and TPJ interacted? Potentially, as participants were deciding whether to transfer money or not, they used ToM to project into the past to cognize about decisions made previously. This result would be in line with the prospection theory of the default model network and theory of mind regions, including the TPJ. If so, this would suggest that coupling between vmPFC and TPJ in this case is not about incorporating others' minds and preferences into strategic decision-making per se, but rather about think about decisions made in the past (as opposed to computations being performed in the present by a computer). Do the authors have evidence against this possibility?

-The reviewer's alternative explanation about using ToM to cognize about past behavior in the Social, but not the Non-social treatment is interesting, but we think that it is an unlikely explanation for the group differences in this experiment for several reasons. First, the participants in the Non-social group were explicitly told that the computer had been programed to draw punishments from a distribution that was based on the behavior of past participants. Therefore, the need to consider past behavior of real people was equated in both treatments. Second, the instructions also stated that the computer was programed in the past and simply responded as programmed on each trial. Lastly, our data here are consistent with the role of TPJ in tracking others current behavior suggested by Carter et al., 2002, Hampton et al., 2008 and Behrens et al., 2008. All three of these studies required participants to consider the behavior of currently acting others. Therefore, while the TPJ may indeed be part of a ToM system involved in projecting into the past to reason about others' beliefs and intentions, we don't believe that it is limited to reasoning about the past or that the tendency to do so should differ in the Social and Non-social treatments.

Second, more description of the behavior of Player B is needed. How did these participants punish? Was there in fact a norm present that participants had to learn? Or was behavioral wildly divergent? Answers to these questions bear on whether participants did indeed need to think about others' preferences and thus the computational role that TPJ is playing.

-The punishment distributions in each treatment are in important detail requested by both reviewers. In order to demonstrate the equality of these distributions clearly, we have decided to add an additional figure (Fig. 2) to the manuscript. This figure shows the best-fit line and standard deviations from linear regression models predicting the punishment amounts at different levels of transfer in both the Social and Non-social treatments. This figure clearly shows that the optimal strategy was to make a fair or even hyper fair transfer because no punishment occurred for transfers of more than 50%.

There were also a few minor issues:

The authors' uncorrected thresholds (p0.001 k=10) were actually quite restrictive, given the small number of trials per subject and the general lack of activation seen in the main GLM. Exploratory analyses at a more lenient threshold, potentially p 0.01 k = 10 or similar, would shed light on whether vmPFC activity during strategic decision-making is influenced by regions other than TPJ.

-While we are happy to include a table of results at a more lenient threshold, we feel that a threshold of p 0.01 and k =10 is likely to report too many false positives given the 10mm FWHM Gaussian smoothing kernel applied during preprocessing. After further consideration, we believe that a more principled approach is to use the smallest individual voxel effect size (t = 2.29) from the TPJ ROI to threshold the whole brain at an uncorrected level. This threshold will reveal any results that are equal to or stronger than the effects in TPJ. This analysis revealed a number of additional regions and the results are now reported in Table 2.

More details are needed about data acquisition. How many runs were acquired and how many trials per run? Similarly, how were trials in the task distributed? Did they vary randomly or were they blocked?

-All trials were acquired during a single functional run, and the order of the trials varied randomly. The text has been revised to clarify this point on page 6, line 122 and page 7, line 143.

On line 111, the authors indicate that "Trials were separated by a fixation cross ITI for approximately 6s." More information would be useful here. Given such a short ITI, the HRF would not have returned to baseline levels by the start of the next trial type screen (which was included in the decision period regressors in the PPI). Thus, unless jittering was included, activity from the previous trial's feedback phase could be impacting the PPI analysis. This may impact how coupling between vmPFC and TPJ is interpreted.

-We have revised and clarified the text to provide additional details about trial timing on page 6, line 119 of the revised manuscript. The details are also copied below for the reviewer's convenience. Briefly, the ITIs between feedback and subsequent choice were a jittered duration of at least 12 seconds.

"Each trial consisted of a treatment screen indicating the trial type for 6s, a participant driven decision period (mean 4.3s, SD 2.7s), then a wait period of 6s followed by a feedback screen displayed for 6s. Trials were separated by a fixation cross ITI for 6 - 8.7s, sampled from a uniform distribution, thus the decision period started at least 12s after the previous trials feedback."

- Line 216: "Figure 3b" should instead be "Figure 2c"

- Line 263: "Fig 3" should be "Fig. 2"

-We have revised the text to correct the errors in labeling the figures.
